# The Influence of Interval Training Combined with Occlusion and Cooling on Selected Indicators of Blood, Muscle Metabolism and Oxidative Stress

**DOI:** 10.3390/jcm12247636

**Published:** 2023-12-12

**Authors:** Bartłomiej Ptaszek, Szymon Podsiadło, Olga Czerwińska-Ledwig, Bartosz Zając, Rafał Niżankowski, Piotr Mika, Aneta Teległów

**Affiliations:** 1 Institute of Applied Sciences, University of Physical Education in Krakow, 31-571 Krakow, Poland; 2 Institute of Clinical Rehabilitation, University of Physical Education in Krakow, 31-571 Krakow, Poland; szymon.podsiadlo@awf.krakow.pl (S.P.); piotr.mika@awf.krakow.pl (P.M.); 3 Institute of Basic Sciences, University of Physical Education in Krakow, 31-571 Krakow, Poland; olga.czerwinska@awf.krakow.pl (O.C.-L.); aneta.teleglow@awf.krakow.pl (A.T.); 4 Laboratory of Functional Diagnostics, Central Scientific and Research Laboratory, University of Physical Education in Krakow, 31-571 Krakow, Poland; bartosz.zajac@awf.krakow.pl; 5 Sano Science, Centre for Computational Medicine, 30-054 Krakow, Poland; rtn@wp.pl

**Keywords:** coagulology, muscle metabolism, oxidative stress, interval training, occlusion, VASPER

## Abstract

There is increasing evidence to support the use of interval training and/or low-impact blood flow restriction exercises in musculoskeletal rehabilitation. The aim of the study was to assess the effect of interval training combined with occlusion and cooling in terms of changes in selected blood parameters affecting the development and progression of atherosclerosis of the lower limbs, as well as selected parameters of muscle metabolism and oxidative stress affecting the growth of muscle mass and regeneration after training. Material and methods: The study included 30 young, healthy and untrained people. The VASPER (Vascular Performance) training system was used—High-Intensity Interval Training with the simultaneous use of occlusion and local cryotherapy. Blood from the project participants was collected six times (2 weeks before the start of training, on the day of training, after the first training, after the 10th training, after the 20th training and two weeks after the end of training). The subjects were randomly divided into three groups: exercises only (controlled), with occlusion and with occlusion and local cryotherapy. Results: Statistical analysis of changes in the average values of indicators in all study groups showed a significant change increase due to the time of testing IGF-1 (F = 2.37, *p* = 0.04), XOD (F = 14.26, *p* = 0.00), D-Dimer (F = 2.90, *p* = 0.02), and decrease in MDA (F = 7.14, *p* = 0.00), T-AOC (F = 11.17, *p* = 0.00), PT Quick (F = 26.37, *p* = 0.00), INR (F = 8.79, *p* = 0.00), TT (F = 3.81, *p* = 0.00). The most pronounced changes were observed in the occlusion and cooling group. Conclusions: Both interval training without and with the modifications used in the study influences coagulation and oxidative stress parameters and, to a small extent, muscle metabolism. It seems reasonable to use occlusion and local cryotherapy in combination with occlusion.

## 1. Introduction

Chronic ischemia of the lower limbs is a condition resulting from insufficient supply of oxygen to the tissues of the lower limbs associated with impaired blood flow in the arteries, caused in 97% of cases by atherosclerosis of the lower limbs. The progressive atherosclerotic process causes narrowing and occlusion of the arterial lumen, consequently leading to chronic ischemia of the lower limbs manifested by pain, shortening of walking distance and intermittent claudication [1]. From a clinical point of view, the finding of atherosclerotic lesions in one place means that it is necessary to take into account the possibility of atherosclerosis also in other vascular locations, which may be associated with the risk of heart attacks and strokes [2]. Conservative and pharmacological treatment includes modification of risk factors such as smoking, dyslipidemia, diabetes, and hypertension and is aimed at stopping the progressive atherosclerotic process [2]. An important and decisive role in the effectiveness of treatment is attributed to regular physical activity, which allows the achievement of the best and most long-lasting effects [3]. The beneficial effects of BFR (blood flow restriction) training were popularized in the 1940s in Japan [4].

Numerous studies have shown that BRF training affects muscle strength, endurance and hypertrophy [5,6,7]. So far, according to the standards of The American College of Sports Medicine, the effective training load that can induce beneficial hypertrophic changes in muscles is 60–100% of 1RM (Rep Max) [5,7]. Recent publications show that this effect can also be achieved by using a low exercise intensity of 20–50% 1RM combined with venous or arterial occlusion, thus avoiding significant damage to muscle fibers [6,8,9,10]. This mechanism is associated with the increased activation of type II FT (Fast Twitching) muscle fibers in oxygen-limited work conditions [11,12].

The effectiveness of exercises combined with occlusion causes significant changes in the level of muscle oxygenation, an increase in anaerobic metabolism products and a significant endocrine response, visible in changes in selected blood parameters [13]. During exercises with occlusion, more than twice the level of lactic acid concentration is exposed, compared to exercises without occlusion [14]. The increased increase in lactic acid levels persists for up to 10 min after the end of exercise, while after approximately 60 min the concentration is still higher than the initial level before the start of the study [9,15]. BRF training also significantly contributes to increasing the concentration of growth hormone (GH), the highest level of which can be observed 20 min after exercise [11,14,16,17,18].

GH plays a key role in preventing atherosclerosis and stimulates the production of peptides called insulin-like growth factors (IGF-1 and IGF-2) [11]. GH and IGF-1 are important mitogenic factors for cardiomyocytes [19]. Their deficiency is associated with a reduced heart mass, decreased late diastolic volume and reduced ejection fraction [19]. The observable increase in the level of VEGF (vascular endothelial growth factor) also plays a major role in the process of angiogenesis and the creation of collateral circulation [20]. Metabolic changes occurring in response to BRF exercises are an important factor influencing the increase in VEGF, which is involved in the process of preventing and developing atherosclerotic lesions.

Pre-cooling of the body applied before training can significantly increase the body’s performance and exercise intensity, both increasing the capacity for prolonged training and the intensity of short-term exercise [21,22]. The pre-cooling mechanism related to the constriction of superficial vessels and probably an increase in blood flow in the muscles generates more efficient elimination of metabolites, reducing the level of DOMS (delayed onset muscle soreness) and lowering the heart rate [21,22].

The aim of the study was to assess the effect of interval training combined with occlusion and cooling in terms of changes in selected blood parameters affecting the development and progression of atherosclerosis of the lower limbs, as well as selected parameters of muscle metabolism and oxidative stress affecting the growth of muscle mass and regeneration after training. Physical exercise combined with occlusion and cooling can be implemented and bring tangible benefits among patients with atherosclerosis of the lower limb arteries, but it can also be widely used in the creation of innovative and effective rehabilitation programs, including patients with chronic ischemia of the lower limbs and other diseases. Analysis of changes in indicators can contribute a lot to science in terms of planning effective therapy.

## 2. Materials and Methods

**Figure 1:**
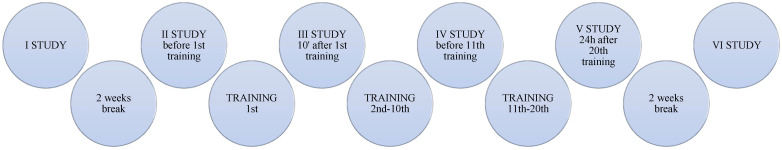
The course of the study.

**Figure 2:**
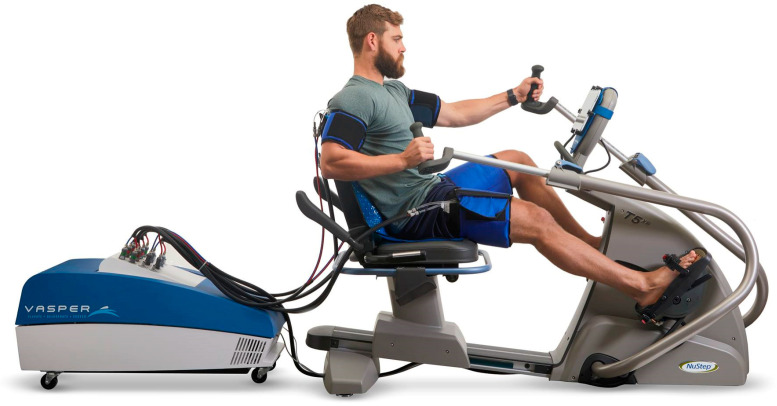
VASPER device (by VASPER Systems LLC).

The research was conducted at the University of Physical Education in Krakow (Poland). The subjects were students who were included in the research program after obtaining medical and physiotherapeutic qualifications. To conduct the experiment, written consent was obtained from the Bioethics Committee at the Regional Medical Chamber in Krakow (164/KBL/OIL/2021). The trials were also registered with the Australian New Zealand Clinical Trials Registry (ACTRN12622000734763). Each volunteer received comprehensive information about the project; if any doubts arose, they had the opportunity to obtain clarification, and then each of them gave informed, written consent to participate in the study (Figure 1). The research covered a group of 30 people, divided into further groups according to the ballot type:

- A total of 10 people performed VASPER Systems LLC (Limited Liability Company) training (Figure 2) without occlusion and cooling (Group CONT)—age [years]: 23.00 ± 0.00; body height [cm]: 164.50 ± 6.44; body mass [kg]: 58.96 ± 10.34.
- A total of 10 people performed VASPER Systems LLC training using occlusion without cooling—the pressure in the occlusion cuffs was 10 mmHg lower than the systolic pressure (Group OCC)—age [years]: 23.22 ± 0.44; body height [cm]: 169.83 ± 9.81; body mass [kg]: 66.31 ± 11.77.
- A total of 10 people performed VASPER Systems LLC training using occlusion and cooling—occlusion as in point 2, additionally combined with activation of the cooling system provided by cuffs and cooling mats located under the feet and in the seat of the subject (Group OCC-COO)—age [years]: 23.25 ± 0.46; body height [cm]: 172.13 ± 9.07; body mass [kg]: 69.45 ± 13.99.

Inclusion criteria: age 20–25, general good health, no comorbidities, no contraindications to participate in HIIT (High-Intensity Interval Training), not engaging in regular physical training, no changes in diet before or during the research.

### 2.1. Analysis of Blood Parameters

Blood for the study was collected from fasting subjects (min. 12 h), from the basilic, cephalic or median vein of the elbow, six times (2 weeks before the start of training, on the day of training, after the first training, after the 10th training, after the 20th training and two weeks after the end of training) using vacuum tubes (for serum testing 6 mL) by a qualified laboratory diagnostician in accordance with applicable protocols. aPTT (Activated Partial Thromboplastin Time), Fibrinogen, D-Dimer, TT (Thrombin Time), PT Quick, and INR (International Normalized Ratio) were determined using the BCS XP analyzer (Siemens Healthcare Diagnostics Product GmbH, Marburg, Germany). CORT (Cortisol) was determined using IMMULITE^®^ 2000 XPi assays (Siemens Healthcare Diagnostics Product GmbH, Marburg, Germany). MIO (Myoglobin) was determined using Alinity™ and STAT Myoglobin Reagent Kit (Abbott, Chicago, IL, USA). HGH (Human Growth Hormone), IGF-1 (Insulin-like Growth Factor 1), VEGF (Vascular Endothelial Growth Factor), MDA (Malondialdehyde), T-AOC (Total Antioxidant Capacity), TOS (Total Oxidative Status) and XOD (Xanthine Oxidase) were determined in the serum. The indices were investigated with photometric tests: HGH—ELISA Kit and IGF-1 600 ELISA Kit (DRG Instruments GmbH, Marburg, Germany); Human VEGF Kit, Human MDA Kit, Human T-AOC Kit, Human TOS Kit, Human XOD Kit (Shanghai Sunred Biological Technology Co., Shanghai, China). The procedure was in accordance with the manufacturer’s recommendations.

### 2.2. Description of the Intervention

Before starting training, each subject underwent an exercise test with lactate measurements (Żołądź Test on the VASPER device), and individual loads not exceeding the lactate threshold were determined. Each participant took part in 20 training sessions (every other day). The training took place at the Functional Diagnostics Laboratory of the University of Physical Education in Krakow.

- OCC-COO group—HIIT training combined with occlusion (on the arms and thighs) and local cooling (built-in cryotherapy system under the feet and under the seat). The pressure in the occlusive cuffs (constant) was 10 mmHg lower than the systolic pressure. The cooling and occlusion system was activated for the entire duration of each training unit. Each session consisted of
  ∘ Introductory part (approx. 2 min)—warm-up, preparation for exercise;
  ∘ Main part (approx. 20 min)—training consisting of 3 × 6 min and 1 min of rest between intervals;
  ∘ Final part (approx. 2 min)—calming down, conscious muscle relaxation;
- Group OCC—like OCC-COO training, with an occlusion variant, but without cooling;
- CONT group—training as in the previous groups, but without the occlusion and cooling variant.

### 2.3. Statistical Analysis

Descriptive statistics were determined: mean (x) as well as standard deviation (SD). The normality of distributions was verified with the Shapiro–Wilk test. Comparisons within and between groups were performed using ANOVA for repeated measures. For comparisons between groups, a multivariate ANOVA was used. If significant changes were observed, post hoc tests were performed. The significance level of *p* = 0.05 was adopted in the analyses. In order to determine sample size, the formula for the minimum sample size was used, in which the confidence interval was 95%, the fraction size of 0.5 and the maximum error of 5% were assumed. The analyses were performed with the use of the Statistica 13 package (Tibco Software Inc., Palo Alto, CA, USA).

## 3. Results

The results are presented in Table 1, Table 2, Table 3, Table 4 and Table 5. Comparisons carried out in individual groups showed statistically significant differences:

- Increase in IGF-1 in the OCC-COO group (F = 2.85, *p* = 0.03), XOD in the OCC-COO group (F = 10.69, *p* = 0.00), OCC group (F = 4.42, *p* = 0.00) and in the CONT group (F = 6.48, *p* = 0.00), fibrinogen in the OCC-COO group (F = 2.81, *p* = 0.03), and D-Dimer in the OCC-COO group (F = 2.66, *p* = 0.04);
- Decrease in MDA in the OCC-COO group (F = 3.74, *p* = 0.01) and CONT group (F = 3.12, *p* = 0.03); T-AOC in the OCC-COO group (F = 3.96, *p* = 0.01), OCC group (F = 7.24, *p* = 0.00) and the CONT group (F = 2.76, *p* = 0.04); PT Quick in the OCC-COO group (F = 17.45, *p* = 0.00), the OCC group (F = 8.27, *p* = 0.00) and CONT group (F = 6.51, *p* = 0.00); INR in the OCC-COO group (F = 3.28, *p* = 0.01), OCC group (F = 4.22, *p* = 0.00) and CONT group (F = 2.67, *p* = 0.05 ); and TT in the OCC-COO group (F = 4.31, *p* = 0.00).

Statistical analysis of changes in the average values of indicators in all study groups showed a significant change increase due to the time of testing IGF-1 (F = 2.37, *p* = 0.04), XOD (F = 14.26, *p* = 0.00), D-Dimer (F = 2.90, *p* = 0.02), and decrease in MDA (F = 7.14, *p* = 0.00), T-AOC (F = 11.17, *p* = 0.00), PT Quick (F = 26.37, *p* = 0.00), INR (F = 8.79, *p* = 0.00), TT (F = 3.81, *p* = 0.00).

**Table 1:** Tested indicators (mean ± standard deviation).

Parameters	Group	I	II	III	IV	V	VI
HGH [ng/mL]	OCC-COO	1.23 ± 0.79	6.64 ± 14.62	6.83 ± 10.51	1.18 ± 1.01	1.41 ± 1.27	1.21 ± 0.78
	OCC	3.64 ± 5.53	4.66 ± 9.64	3.45 ± 4.86	7.02 ± 12.90	2.05 ± 3.29	3.53 ± 7.93
	CONT	2.06 ± 1.25	1.27 ± 0.78	1.15 ± 0.88	1.42 ± 1.13	2.46 ± 2.70	2.04 ± 2.82
IGF-1 [ng/mL]	OCC-COO	84.27 ± 17.11	83.49 ± 20.48	88.97 ± 22.16	95.18 ± 49.15	147.50 ± 75.62	117.90 ± 43.82
	OCC	85.35 ± 11.71	96.27 ± 28.76	94.96 ± 37.09	116.05 ± 62.60	117.54 ± 42.13	131.81 ± 60.50
	CONT	120.76 ± 50.52	92.89 ± 39.38	105.85 ± 29.30	147.61 ± 49.20	99.78 ± 51.50	115.35 ± 65.21
MDA [nmol/mL]	OCC-COO	4.30 ± 1.81	4.06 ± 1.83	3.45 ± 2.27	2.75 ± 1.13	3.60 ± 1.92	2.73 ± 1.80
	OCC	3.84 ± 1.16	3.43 ± 1.56	3.32 ± 1.01	2.45 ± 1.04	3.26 ± 1.42	2.89 ± 1.24
	CONT	4.55 ± 1.86	3.57 ± 1.12	3.24 ± 0.95	3.44 ± 1.08	2.86 ± 1.85	2.85 ± 1.04
T-AOC [U/mL]	OCC-COO	6.91 ± 3.98	6.88 ± 2.25	7.57 ± 3.34	3.98 ± 1.88	4.00 ± 1.25	5.91 ± 3.17
	OCC	5.87 ± 3.48	7.61 ± 2.37	6.07 ± 2.90	3.61 ± 1.78	4.62 ± 2.76	3.15 ± 2.20
	CONT	6.14 ± 1.51	8.84 ± 5.60	7.35 ± 5.39	3.55 ± 1.33	4.67 ± 2.25	3.33 ± 1.61
TOS [U/mL]	OCC-COO	25.97 ± 9.03	30.07 ± 17.30	31.71 ± 13.43	25.96 ± 6.51	36.36 ± 20.72	37.56 ± 17.75
	OCC	24.38 ± 13.95	33.41 ± 18.83	39.92 ± 20.17	63.33 ± 101.49	68.12 ± 102.31	25.53 ± 6.66
	CONT	40.51 ± 14.44	31.35 ± 9.56	28.39 ± 11.78	31.97 ± 8.43	33.84 ± 5.00	81.06 ± 142.98
VEGF [ng/L]	OCC-COO	857.52 ± 258.86	837.71 ± 281.89	745.30 ± 285.13	953.71 ± 395.19	911.27 ± 424.97	834.30 ± 239.77
	OCC	891.03 ± 265.45	997.31 ± 349.36	744.82 ± 268.71	1183.08 ± 681.44	976.39 ± 442.41	900.97 ± 436.82
	CONT	753.57 ± 162.34	906.42 ± 380.74	830.65 ± 223.52	861.13 ± 280.11	800.30 ± 116.45	985.13 ± 375.70
XOD [ng/mL]	OCC-COO	17.18 ± 3.82	13.06 ± 3.53	14.16 ± 1.93	25.96 ± 10.02	21.21 ± 5.63	20.07 ± 3.50
	OCC	15.82 ± 3.08	16.50 ± 5.08	12.98 ± 1.86	21.94 ± 9.05	24.93 ± 11.62	21.79 ± 8.77
	CONT	16.23 ± 3.87	14.73 ± 4.19	13.76 ± 2.55	19.18 ± 4.87	24.22 ± 5.96	16.70 ± 4.61
CORT [μg/dL]	OCC-COO	18.10 ± 4.71	18.61 ± 2.40	16.32 ± 2.31	17.52 ± 1.79	17.63 ± 1.44	18.19 ± 1.65
	OCC	19.72 ± 4.19	19.04 ± 5.49	18.38 ± 2.30	18.09 ± 2.69	18.37 ± 2.81	17.57 ± 2.49
	CONT	19.42 ± 4.78	20.05 ± 3.21	18.08 ± 4.13	21.12 ± 2.98	20.75 ± 5.14	19.72 ± 4.10
MIO [μg/L]	OCC-COO	40.13 ± 29.22	31.79 ± 8.43	37.01 ± 18.04	34.76 ± 13.06	34.07 ± 14.92	32.71 ± 8.48
	OCC	40.99 ± 19.57	36.98 ± 14.65	37.22 ± 17.62	34.01 ± 13.41	28.53 ± 9.47	60.69 ± 86.90
	CONT	25.67 ± 6.12	29.63 ± 12.63	28.83 ± 12.27	36.40 ± 23.88	27.87 ± 5.72	33.45 ± 18.23
PT Quick [%]	OCC-COO	99.41 ± 9.63	100.87 ± 7.18	95.25 ± 8.20	90.96 ± 7.70	90.94 ± 8.83	89.03 ± 7.30
	OCC	104.45 ± 10.80	104.60 ± 14.94	99.58 ± 16.48	91.73 ± 16.70	95.70 ± 14.19	95.81 ± 16.20
	CONT	101.20 ± 8.82	101.37 ± 11.00	97.80 ± 6.09	90.38 ± 6.91	93.97 ± 8.59	91.58 ± 9.39
INR	OCC-COO	1.12 ± 0.07	1.08 ± 0.05	1.09 ± 0.05	1.12 ± 0.05	1.12 ± 0.06	1.10 ± 0.05
	OCC	1.09 ± 0.07	1.06 ± 0.08	1.07 ± 0.11	1.13 ± 0.12	1.10 ± 0.09	1.06 ± 0.08
	CONT	1.11 ± 0.06	1.08 ± 0.06	1.08 ± 0.04	1.13 ± 0.05	1.10 ± 0.06	1.08 ± 0.06
aPTT [s]	OCC-COO	29.40 ± 1.72	29.80 ± 1.80	29.88 ± 2.45	29.42 ± 1.32	29.87 ± 1.75	29.39 ± 1.77
	OCC	28.17 ± 2.87	28.50 ± 3.13	29.12 ± 3.68	28.73 ± 3.62	28.73 ± 4.00	28.42 ± 3.21
	CONT	27.98 ± 1.11	28.07 ± 1.89	28.53 ± 1.03	28.37 ± 0.70	27.65 ± 1.48	27.62 ± 1.69
Fibrinogen [g/L]	OCC-COO	2.32 ± 0.27	2.30 ± 0.51	2.15 ± 0.32	2.78 ± 0.83	2.39 ± 0.29	2.29 ± 0.20
	OCC	2.78 ± 0.66	2.55 ± 0.41	2.43 ± 0.27	2.44 ± 0.53	2.51 ± 0.69	2.71 ± 0.82
	CONT	2.76 ± 0.69	2.69 ± 0.84	2.57 ± 0.69	2.85 ± 0.95	2.77 ± 0.76	2.63 ± 0.68
D-Dimer [mg/L]	OCC-COO	0.20 ± 0.04	0.23 ± 0.08	0.28 ± 0.18	0.20 ± 0.06	0.38 ± 0.24	0.28 ± 0.21
	OCC	0.28 ± 0.17	0.28 ± 0.19	0.24 ± 0.13	0.24 ± 0.13	0.29 ± 0.20	0.25 ± 0.13
	CONT	0.27 ± 0.10	0.30 ± 0.14	0.27 ± 0.11	0.26 ± 0.11	0.34 ± 0.17	0.29 ± 0.18
TT [s]	OCC-COO	18.12 ± 0.79	18.39 ± 1.14	18.80 ± 0.86	17.22 ± 1.21	18.79 ± 0.77	18.07 ± 0.99
	OCC	17.62 ± 1.23	18.08 ± 1.21	18.20 ± 1.07	18.29 ± 1.44	18.50 ± 1.43	17.73 ± 1.07
	CONT	17.30 ± 1.39	17.77 ± 1.16	17.90 ± 0.88	17.45 ± 1.58	18.42 ± 1.21	17.85 ± 0.95

HGH (Human Growth Hormone), IGF-1 (Insulin-like Growth Factor 1), VEGF (Vascular Endothelial Growth Factor), MDA (Malondialdehyde), T-AOC (Total Antioxidant Capacity), TOS (Total Oxidative Status), XOD (Xanthine Oxidase), CORT (Cortisol), MIO (Myoglobin), PT Quick, INR (International Normalized Ratio), aPTT (Activated Partial Thromboplastin Time), TT (Thrombin Time).

**Table 2:** Analysis of variance for repeated measurements for the studied indicators in groups—F test value and significance level *p*.

Parameters	Groups	F Test Value	Significance Level *p*
HGH [ng/mL]	OCC-COO	1.58	0.19
	OCC	0.84	0.53
	CONT	0.65	0.66
IGF-1 [ng/mL]	OCC-COO	2.85	0.03
	OCC	1.44	0.23
	CONT	0.96	0.46
MDA [nmol/mL]	OCC-COO	3.74	0.01
	OCC	2.30	0.06
	CONT	3.12	0.03
T-AOC [U/mL]	OCC-COO	3.96	0.01
	OCC	7.24	0.00
	CONT	2.76	0.04
TOS [U/mL]	OCC-COO	1.30	0.28
	OCC	1.11	0.37
	CONT	0.70	0.63
VEGF [ng/L]	OCC-COO	0.57	0.72
	OCC	1.17	0.34
	CONT	0.78	0.57
XOD [ng/mL]	OCC-COO	10.69	0.00
	OCC	4.42	0.00
	CONT	6.48	0.00
CORT [μg/dL]	OCC-COO	0.94	0.46
	OCC	0.94	0.46
	CONT	2.11	0.10
MIO [μg/L]	OCC-COO	0.40	0.84
	OCC	0.85	0.53
	CONT	1.03	0.42
PT Quick [%]	OCC-COO	17.45	0.00
	OCC	8.27	0.00
	CONT	6.51	0.00
INR	OCC-COO	3.28	0.01
	OCC	4.22	0.00
	CONT	2.67	0.05
aPTT [s]	OCC-COO	0.59	0.71
	OCC	1.36	0.26
	CONT	0.85	0.53
Fibrinogen [g/L]	OCC-COO	2.81	0.03
	OCC	0.97	0.45
	CONT	0.41	0.84
D-Dimer [mg/L]	OCC-COO	2.66	0.04
	OCC	0.57	0.72
	CONT	1.26	0.31
TT [s]	OCC-COO	4.31	0.00
	OCC	1.77	0.14
	CONT	0.73	0.61

HGH (Human Growth Hormone), IGF-1 (Insulin-like Growth Factor 1), VEGF (Vascular Endothelial Growth Factor), MDA (Malondialdehyde), T-AOC (Total Antioxidant Capacity), TOS (Total Oxidative Status), XOD (Xanthine Oxidase), CORT (Cortisol), MIO (Myoglobin), PT Quick, INR (International Normalized Ratio), aPTT (Activated Partial Thromboplastin Time), TT (Thrombin Time). ANOVA test for repeated measurements was performed; significance level of *p* = 0.05.

**Table 3:** Post hoc test values for the studied indicators depending on the study in the same groups.

Parameters	Study	I	II	III	IV	V	VI
IGF-1 [ng/mL] OCC-COO	I		0.97	0.83	0.61	0.00	0.12
	II	0.97		0.80	0.58	0.00	0.11
	III	0.83	0.80		0.77	0.01	0.18
	IV	0.61	0.58	0.77		0.02	0.29
	V	0.00	0.00	0.01	0.02		0.17
	VI	0.12	0.11	0.18	0.29	0.17	
MDA [nmol/mL] OCC-COO	I		0.62	0.08	0.00	0.15	0.00
	II	0.62		0.21	0.01	0.33	0.01
	III	0.08	0.21		0.15	0.76	0.14
	IV	0.00	0.01	0.15		0.08	0.98
	V	0.15	0.33	0.76	0.08		0.08
	VI	0.00	0.01	0.14	0.98	0.08	
MDA [nmol/mL] CONT	I		0.06	0.02	0.04	0.00	0.00
	II	0.06		0.51	0.79	0.17	0.16
	III	0.02	0.51		0.69	0.46	0.44
	IV	0.04	0.79	0.69		0.26	0.25
	V	0.00	0.17	0.46	0.26		0.98
	VI	0.00	0.16	0.44	0.25	0.98	
T-AOC [U/mL] OCC-COO	I		0.98	0.55	0.01	0.01	0.37
	II	0.98		0.53	0.01	0.01	0.38
	III	0.55	0.53		0.00	0.00	0.14
	IV	0.01	0.01	0.00		0.99	0.09
	V	0.01	0.01	0.00	0.99		0.09
	VI	0.37	0.38	0.14	0.09	0.09	
T-AOC [U/mL] OCC	I		0.06	0.83	0.01	0.16	0.00
	II	0.06		0.09	0.00	0.00	0.00
	III	0.83	0.09		0.01	0.11	0.00
	IV	0.01	0.00	0.01		0.26	0.61
	V	0.16	0.00	0.11	0.26		0.10
	VI	0.00	0.00	0.00	0.61	0.10	
T-AOC [U/mL] CONT	I		0.16	0.52	0.18	0.44	0.14
	II	0.16		0.43	0.01	0.04	0.01
	III	0.52	0.43		0.05	0.16	0.04
	IV	0.18	0.01	0.05		0.55	0.90
	V	0.44	0.04	0.16	0.55		0.48
	VI	0.14	0.01	0.04	0.90	0.48	
XOD [ng/mL] OCC-COO	I		0.05	0.15	0.00	0.06	0.17
	II	0.05		0.60	0.00	0.00	0.00
	III	0.15	0.60		0.00	0.00	0.01
	IV	0.00	0.00	0.00		0.03	0.01
	V	0.06	0.00	0.00	0.03		0.59
	VI	0.17	0.00	0.01	0.01	0.59	
XOD [ng/mL] OCC	I		0.83	0.36	0.05	0.01	0.06
	II	0.83		0.26	0.08	0.01	0.09
	III	0.36	0.26		0.01	0.00	0.01
	IV	0.05	0.08	0.01		0.34	0.96
	V	0.01	0.01	0.00	0.34		0.31
	VI	0.06	0.09	0.01	0.96	0.31	
XOD [ng/mL] CONT	I		0.48	0.25	0.17	0.00	0.82
	II	0.48		0.65	0.05	0.00	0.36
	III	0.25	0.65		0.02	0.00	0.17
	IV	0.17	0.05	0.02		0.02	0.25
	V	0.00	0.00	0.00	0.02		0.00
	VI	0.82	0.36	0.17	0.25	0.00	
PT Quick [%] OCC-COO	I		0.38	0.02	0.00	0.00	0.00
	II	0.38		0.00	0.00	0.00	0.00
	III	0.02	0.00		0.01	0.01	0.00
	IV	0.00	0.00	0.01		0.99	0.25
	V	0.00	0.00	0.01	0.99		0.26
	VI	0.00	0.00	0.00	0.25	0.26	
PT Quick [%] OCC	I		0.95	0.06	0.00	0.00	0.00
	II	0.95		0.06	0.00	0.00	0.00
	III	0.06	0.06		0.00	0.14	0.15
	IV	0.00	0.00	0.00		0.13	0.12
	V	0.00	0.00	0.14	0.13		0.96
	VI	0.00	0.00	0.15	0.12	0.96	
PT Quick [%] CONT	I		0.95	0.21	0.00	0.01	0.00
	II	0.95		0.19	0.00	0.01	0.00
	III	0.21	0.19		0.01	0.16	0.03
	IV	0.00	0.00	0.01		0.19	0.65
	V	0.01	0.01	0.16	0.19		0.38
	VI	0.00	0.00	0.03	0.65	0.38	
INR OCC-COO	I		0.01	0.06	1.00	1.00	0.08
	II	0.01		0.32	0.01	0.01	0.25
	III	0.06	0.32		0.06	0.06	0.88
	IV	1.00	0.01	0.06		1.00	0.08
	V	1.00	0.01	0.06	1.00		0.08
	VI	0.08	0.25	0.88	0.08	0.08	
INR OCC	I		0.11	0.42	0.04	0.66	0.11
	II	0.11		0.42	0.00	0.04	1.00
	III	0.42	0.42		0.00	0.21	0.42
	IV	0.04	0.00	0.00		0.10	0.00
	V	0.66	0.04	0.21	0.10		0.04
	VI	0.11	1.00	0.42	0.00	0.04	
INR CONT	I		0.09	0.06	0.34	0.70	0.13
	II	0.09		0.85	0.01	0.19	0.85
	III	0.06	0.85		0.01	0.13	0.70
	IV	0.34	0.01	0.01		0.19	0.02
	V	0.70	0.19	0.13	0.19		0.26
	VI	0.13	0.85	0.70	0.02	0.26	
Fibrinogen [g/L] OCC-COO	I		0.94	0.36	0.01	0.69	0.88
	II	0.94		0.40	0.01	0.64	0.94
	III	0.36	0.40		0.00	0.19	0.45
	IV	0.01	0.01	0.00		0.04	0.01
	V	0.69	0.64	0.19	0.04		0.58
	VI	0.88	0.94	0.45	0.01	0.58	
D-Dimer [mg/L] OCC-COO	I		0.60	0.18	0.99	0.00	0.19
	II	0.60		0.40	0.59	0.02	0.43
	III	0.18	0.40		0.17	0.10	0.97
	IV	0.99	0.59	0.17		0.00	0.18
	V	0.00	0.02	0.10	0.00		0.10
	VI	0.19	0.43	0.97	0.18	0.10	
TT [s] OCC-COO	I		0.51	0.10	0.03	0.10	0.89
	II	0.51		0.31	0.01	0.32	0.42
	III	0.10	0.31		0.00	0.98	0.07
	IV	0.03	0.01	0.00		0.00	0.04
	V	0.10	0.32	0.98	0.00		0.08
	VI	0.89	0.42	0.07	0.04	0.08	

IGF-1 (Insulin-like Growth Factor 1), MDA (Malondialdehyde), T-AOC (Total Antioxidant Capacity), XOD (Xanthine Oxidase), PT Quick, INR (International Normalized Ratio), TT (Thrombin Time). NIR post hoc test was performed; significance level of *p* = 0.05.

**Table 4:** Multivariate analysis of variance for repeated measurements for the studied indicators between groups—F test value and significance level *p* depending on the factor used.

Parameters	Factor	F Test Value	Significance Level *p*
HGH [ng/mL]	S	0.66	0.66
	I	0.45	0.65
	S*I	1.20	0.30
IGF-1 [ng/mL]	S	2.37	0.04
	I	0.69	0.51
	S*I	1.22	0.29
MDA [nmol/mL]	S	7.14	0.00
	I	0.12	0.88
	S*I	0.81	0.62
T-AOC [U/mL]	S	11.17	0.00
	I	0.34	0.72
	S*I	1.01	0.44
TOS [U/mL]	S	0.69	0.63
	I	0.50	0.62
	S*I	1.14	0.34
VEGF [ng/L]	S	1.30	0.27
	I	0.59	0.57
	S*I	0.53	0.87
XOD [ng/mL]	S	14.26	0.00
	I	0.29	0.75
	S*I	1.32	0.23
CORT [μg/dL]	S	1.60	0.17
	I	1.15	0.34
	S*I	0.94	0.50
MIO [μg/L]	S	0.64	0.67
	I	0.75	0.48
	S*I	0.69	0.73
PT Quick [%]	S	26.37	0.00
	I	0.37	0.70
	S*I	0.49	0.89
INR	S	8.79	0.00
	I	0.29	0.75
	S*I	0.54	0.86
aPTT [s]	S	1.75	0.13
	I	0.90	0.42
	S*I	0.48	0.90
Fibrinogen [g/L]	S	1.51	0.19
	I	1.00	0.38
	S*I	1.20	0.30
D-Dimer [mg/L]	S	2.90	0.02
	I	0.09	0.91
	S*I	1.06	0.40
TT [s]	S	3.81	0.00
	I	0.56	0.58
	S*I	1.20	0.30

HGH (Human Growth Hormone), IGF-1 (Insulin-like Growth Factor 1), VEGF (Vascular Endothelial Growth Factor), MDA (Malondialdehyde), T-AOC (Total Antioxidant Capacity), TOS (Total Oxidative Status), XOD (Xanthine Oxidase), CORT (Cortisol), MIO (Myoglobin), PT Quick, INR (International Normalized Ratio), aPTT (Activated Partial Thromboplastin Time), TT (Thrombin Time), S-Study, I-Intervention, S*I—Study*Intervention. Multivariate ANOVA test was performed; significance level of *p* = 0.05.

**Table 5:** Post hoc test values for the studied indicators depending on the study between groups.

Parameters	Study	I	II	III	IV	V	VI
IGF-1 [ng/mL]	I		0.81	0.90	0.09	0.02	0.03
	II	0.81		0.71	0.05	0.01	0.02
	III	0.90	0.71		0.12	0.03	0.04
	IV	0.09	0.05	0.12		0.53	0.63
	V	0.02	0.01	0.03	0.53		0.89
	VI	0.03	0.02	0.04	0.63	0.89	
MDA [nmol/mL]	I		0.08	0.00	0.00	0.00	0.00
	II	0.08		0.20	0.00	0.13	0.00
	III	0.00	0.20		0.05	0.82	0.06
	IV	0.00	0.00	0.05		0.09	0.97
	V	0.00	0.13	0.82	0.09		0.09
	VI	0.00	0.00	0.06	0.97	0.09	
T-AOC [U/mL]	I		0.06	0.37	0.00	0.01	0.00
	II	0.06		0.33	0.00	0.00	0.00
	III	0.37	0.33		0.00	0.00	0.00
	IV	0.00	0.00	0.00		0.35	0.48
	V	0.01	0.00	0.00	0.35		0.81
	VI	0.00	0.00	0.00	0.48	0.81	
XOD [ng/mL]	I		0.27	0.06	0.00	0.00	0.02
	II	0.27		0.44	0.00	0.00	0.00
	III	0.06	0.44		0.00	0.00	0.00
	IV	0.00	0.00	0.00		0.69	0.06
	V	0.00	0.00	0.00	0.69		0.02
	VI	0.02	0.00	0.00	0.06	0.02	
PT Quick [%]	I		0.62	0.00	0.00	0.00	0.00
	II	0.62		0.00	0.00	0.00	0.00
	III	0.00	0.00		0.00	0.00	0.00
	IV	0.00	0.00	0.00		0.07	0.40
	V	0.00	0.00	0.00	0.07		0.34
	VI	0.00	0.00	0.00	0.40	0.34	
INR	I		0.00	0.01	0.06	0.90	0.01
	II	0.00		0.30	0.00	0.00	0.46
	III	0.01	0.30		0.00	0.01	0.76
	IV	0.06	0.00	0.00		0.08	0.00
	V	0.90	0.00	0.01	0.08		0.00
	VI	0.01	0.46	0.76	0.00	0.00	
D-Dimer [mg/L]	I		0.50	0.56	0.53	0.00	0.42
	II	0.50		0.93	0.20	0.02	0.88
	III	0.56	0.93		0.23	0.01	0.82
	IV	0.53	0.20	0.23		0.00	0.15
	V	0.00	0.02	0.01	0.00		0.03
	VI	0.42	0.88	0.82	0.15	0.03	
TT [s]	I		0.13	0.02	0.85	0.00	0.54
	II	0.13		0.37	0.09	0.07	0.37
	III	0.02	0.37		0.01	0.36	0.07
	IV	0.85	0.09	0.01		0.00	0.42
	V	0.00	0.07	0.36	0.00		0.01
	VI	0.54	0.37	0.07	0.42	0.01	

IGF-1 (Insulin-like Growth Factor 1), MDA (Malondialdehyde), T-AOC (Total Antioxidant Capacity), XOD (Xanthine Oxidase), PT Quick, INR (International Normalized Ratio), TT (Thrombin Time). NIR post hoc test was performed; significance level of *p* = 0.05.

## 4. Discussion

Physical exercise, mainly aerobic, is a well-researched method of strengthening the capillary network in skeletal muscles [23]. There is also evidence that high-intensity resistance exercise also induces angiogenic signaling in activated muscles [24]. An increase in VEGF levels, which is considered an important modulator of vasculogenesis and angiogenesis, is necessary for hypertrophy and can be stimulated by lactate accumulation and hypoxia [25,26,27,28]. There is also evidence of an increase in the rate of angiogenesis when lactate levels are increased, thus producing an even greater hypertrophic stimulus [29]. Accordingly, lactate accumulation resulting from occlusion-induced hypoxia is a stimulus for muscle hypertrophy. Larkin et al. (2012) assessed the post-exercise VEGF level in serum during unilateral knee extension with and without the addition of BFR. They also examined mRNA transcripts indicating angiogenesis, i.e.,: VEGF and its main receptor (VEGF-R2), hypoxia-inducible factor 1 alpha (HIF-1alpha) and nitric oxide synthase isoforms (NOS) [30]. The key finding was that the BFR condition induced a significantly greater angiogenic response compared with the non-BFR condition. Although serum VEGF levels did not differ significantly between groups, mRNA transcripts for VEGF, VEGF-R2, and neuronal nitric oxide synthase were significantly greater at BFR at 4 and 24 h postexercise. In our studies, we observed trends towards lower VEGF 24 h after the first training, and higher concentrations only after the 10th and 20th training, but these changes were not statistically significant.

Muscle hypertrophy and increased strength after a single high-intensity exercise session are thought to be related to the recruitment of high-threshold motor units [31,32]. The recruitment of these motor units results in a significant increase in mechanical stress [33,34] and endocrine responses [35], as well as the accumulation of metabolites [36]. It is hypothesized that the accumulation of metabolic byproducts and/or the hypoxia-induced stimulation of afferent nerve fibers causes an increase in the secretion of GH and GH-releasing hormone [37]. Kraemer and Ratamess (2005) found that a large, sharp increase in GH after exercise stimulates IGF secretion, leading to increased protein synthesis and ultimately muscle hypertrophy [38]. Pierce et al. (2006) observed a nine-fold increase in serum GH concentration from baseline to the cessation of knee extension exercises using BFR [39]. There are also studies that report increases in GH up to 290 times the baseline [14,40,41,42], with the GH response after BFR and low-intensity exercise being similar or even higher than that observed during high-intensity exercise [38,43]. It has been suggested that lactate accumulation plays a key role in GH release during exercise, which is supported by the fact that people lacking the myophosphorylase enzyme (those who do not show an increase in blood lactate levels during exercise) show a weaker GH response [44]. However, Reeves et al. (2006) demonstrated an increased GH response in the BFR training group compared to traditional non-BFR training groups with the same lactate concentrations [45], suggesting additional mechanisms. IGF-1 production also increases with GH. In fact, IGF-1 increases protein synthesis and activates satellite cells, which causes myofibril hypertrophy [46]. Furthermore, muscle hypertrophy occurred with the viral overexpression of IGF-1 [47]. The increase in IGF1 observed in some studies may be related to hemoconcentration due to changes in plasma volume after BFR resistance exercise [48]. After a training series, a progressive increase in IGF1 levels was observed after 2 weeks of twice-daily BFR exercise [49]. Therefore, the overall relationship between BFR resistance exercise and the GH–IGF1 axis remains controversial. West et al. (2009) reported no increase in muscle protein synthesis or the phosphorylation of signaling proteins after resistance exercise with elevated systemic concentrations of T, GH, and IGF1 compared to low systemic concentrations of the same anabolic hormones [50]. An important mechanism underlying BFR-induced hypertrophy may be the prolonged duration of metabolic acidosis, which induces systemic GH release. Abe et al. (2005) reported an increased IGF-1 response to low-intensity resistance training with BFR. The control group in this study performed a protocol of the same intensity and volume but did not show a similar response [49]. In our studies, we observed a statistically significant increase in IGF-1 after completed training in the occlusion and cooling group; in the occlusion-only group, an increasing tendency was also observed, although statistically insignificant. When analyzing HGH, we did not note any statistically significant changes.

The stress of resistance exercise is known to increase cortisol levels. Fujita et al. (2007) compared four sets of exercises with and without BFR. The authors found increased cortisol concentration after the BFR protocol compared to the control session. In this study, higher cortisol levels after the BFR session likely indicated a stronger stress response; this is evident by the fact that cortisol levels returned to baseline values approximately 1 h after exercise [51]. In our study, we did not observe changes in CORT over time or differences between groups.

The effects of resistance exercise combined with occlusion on systemic physiological markers of muscle damage are not well understood. Although no increased levels of creatine kinase and myoglobin were observed after two sets of resistance exercises with BFR [14,51], there are studies that report rhabdomyolysis [52]. In addition to the potential for muscle damage during BFR training, there is a hypothetical risk of microvascular dysfunction due to reperfusion, which occurs when blood flow is restored after a period of stenosis or ischemia [53]. It is hypothesized that during occlusion, a hypoxic and ischemic muscle environment is created, which causes high levels of metabolic stress as well as mechanical stress when BFR is used in conjunction with exercise. Both metabolic stress and mechanical stress have been described as “major factors of hypertrophy” [54] and have been assumed to activate other mechanisms to induce muscle growth: cell swelling [55], increased systemic hormone production [6,45], the production of reactive oxygen species (ROS) [51,56], the increased recruitment of fast-twitch fibers [57,58] and the intramuscular transmission of anabolic/anti-catabolic signals [18,41,59]. Additionally, although not statistically significant, Goldfarb et al. (2008) showed that the ratio of protein carbonyls and glutathione (systemic indicators of oxidative stress) increased almost twofold after BFR resistance exercise in a small group of men [60]. Analyzing the oxidative stress indicators in our subjects, we observed a decrease in MDA, statistically significant only in the CONT group, an increase in XOD in all groups after 10 and 20 training sessions and a decrease in T-AOC in all groups after 10 and 20 training sessions without a simultaneous change in TOS.

To the best of our knowledge, this study is the first to evaluate the effects of interval training combined with occlusion or occlusion and cooling on indices of muscle metabolism and oxidative stress in young healthy humans.

Study Limitation: The current experiment was not without flaws related to the lack of a uniform diet and its monitoring (the inclusion criterion was only the lack of change in diet before and during the project), but it undoubtedly showed the impact of interval training and its modifications on the tested indicators. Research should continue in groups of patients divided by gender and be adapted for people with vascular diseases and athletes.

## 5. Conclusions

Based on the research conducted, the following conclusions can be drawn:

- Changes in the examined indicators (IGF-1, MDA, T-AOC, XOD, PT Quick, INR, Fibrinogen, D-Dimer, and TT) were observed after a series of training sessions, not after a single training unit.
- Both interval training without and with the modifications used in the study influence coagulation (PT Quick, INR, Fibrinogen, D-Dimer, and TT) and oxidative stress (MDA, T-AOC, and XOD) parameters and, to a small extent, muscle metabolism (IGF-1).
- It seems reasonable to use occlusion and local cryotherapy in combination with occlusion.

## Data Availability

All data generated or analyzed during this study are included in this published article.
